# OligoR: A Native HDX/MS Data Processing Application Dedicated to Oligonucleotides

**DOI:** 10.1021/acs.analchem.3c01321

**Published:** 2023-06-13

**Authors:** Eric Largy, Matthieu Ranz

**Affiliations:** CNRS, INSERM, ARNA, UMR 5320, U1212, IECB, Univ. Bordeaux, F-33600 Pessac, France

## Abstract

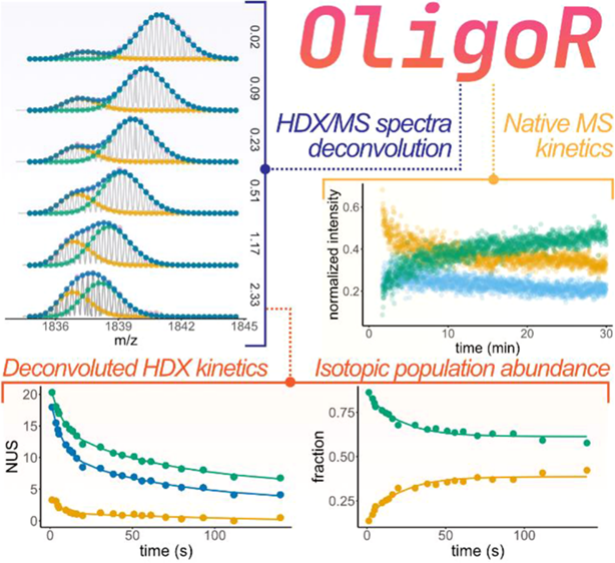

Hydrogen–deuterium exchange mass spectrometry (HDX/MS) is increasingly used to study the dynamics of protein conformation. Coupled to native MS, HDX can also characterize the conformations of oligonucleotides and their binding to cations, small molecules, and proteins. Data processing and visualization of native HDX/MS of oligonucleotides requires dedicated software solutions. OligoR is a web-browser-based application that addresses the specific needs of DNA HDX/MS and native MS experiments from raw data in an open format to visualization and export of results. Whole experiments spanning many time points can be processed in minutes for several mass-separated species. To access valuable folding dynamics information, we have developed a simple and robust approach to deconvolute bimodal isotope distributions, even when they are highly overlapping. This approach is based on modeling physically possible isotope distributions determined from chemical formulae and could be extended to any type of analyte (proteins, peptides, sugars, and small molecules). All results are presented in interactive data tables, and publication-quality figures can be generated, customized, and exported.

## Introduction

Hydrogen–deuterium exchange mass spectrometry (HDX/MS) is a technique of choice to study the structural dynamics of proteins.^1−5^ Incubation of a protein in a deuterated buffer results in the exchange of amide hydrogen by deuterium isotopes at a rate that strongly depends on the hydrogen-bonding status and solvent accessibility.^6−8^ Therefore, the deuteration yields inform on the structure of proteins. HDX/MS is increasingly used both in academic and industrial contexts to compare different states of a protein in, e.g., epitope mapping,^2,9,10^ batch-to-batch comparisons,^11^ biosimilarity evaluation,^2,12^ and stress testing.^13^

Most HDX/MS experiments share a similar bottom-up approach: the protein is first incubated in a deuterium-rich buffer for a defined time, then the reaction is quenched, the protein denatured and digested with a protease, and the resulting peptides are finally analyzed by LC/MS.^8^ The deuterium content is therefore quantified for many peptides, over several time points, generating a large amount of data.^14,15^ Consequently, several programs have been developed to assist in HDX/MS raw data processing (peptide detection and identification, determination of their deuterium content, isotopic distribution modeling, and fitting),^16−26^ post-processing (comparison of deuterium uptake across samples and assessment of statistical significance), and visualization.^19,22,24,27−35^

We have recently demonstrated that HDX/MS can also apply to DNA oligonucleotides.^36^ Such an approach can characterize the secondary structures of oligonucleotides (e.g., genomic and viral sequences, aptamers), investigate complicated folding pathways, and probe oligonucleotide complexes with cations, small molecules, and proteins. The analytical workflow we propose differs from that of routine protein HDX/MS: the analytes are injected in the mass spectrometer with no quenching or denaturation steps and thus without chromatographic separation. We have employed the following two approaches.

Real-time monitoring of the exchange (called “RT-HDX”): the exchange is triggered by manual mixing of a pre-deuterated DNA solution with a non-deuterated buffer. The exchanging solution is infused in the mass spectrometer and analyzed in a time-dependent manner: each MS scan corresponds to a different deuteration time point.
Continuous-flow mixing (called “CF-HDX”): the pre-deuterated analytes are exchanged for a defined time by mixing with a non-deuterated buffer from a mixing tee to the mass spectrometer. The duration of the exchange is controlled by the volume and the flow rate between the mixing tee and the mass spectrometer. All MS scans correspond to the same deuteration time point.

This has several consequences, which are listed below.

(i) The MS measurement can be performed in native conditions, and therefore, several species (including non-covalent complexes) can be mass-separated, and their deuteration quantified concurrently.
(ii) The number of analytes in each injection is reduced compared to bottom-up approaches (1–10 vs hundreds), but all analytes to be considered are present in all scans (no chromatographic separation).
(iii) The number of time points is increased compared to bottom-up approaches (thousands for RT-HDX vs 1–10 for bottom-up).
(iv) Theoretical isotopic envelopes must be calculated for DNA analytes, whose number of exchangeable sites (one for dT, two for dA and dC, and three for dG) differs from that of peptides (one per amide, except prolines). Non-covalent binding of cations and organic molecules must also be considered.

An application specifically designed to process oligonucleotide HDX/MS raw data is therefore necessary to handle these constraints. Further analysis of the processed data is also desirable: fitting of the exchange kinetics is necessary to determine exchange rates and unlock underlying folding kinetics and equilibrium constants. Some oligonucleotide conformations exchange simultaneously through different kinetics regimes (the EX1 and EX2 mechanisms). EX1 kinetics and multiple conformers interconverting slower than their respective exchange rates yield bimodal distributions.^21,37^ We want to deconvolute these distributions to access the exchange rates of individual populations, as well as the rate of unfolding of the analyte to less-folded EX1-competent conformations. Deconvolution of deuterated isotopic distributions has only been achieved in a few of the available HDX/MS software programs and is only functioning for peptides.

We introduce here an open-source R-based application, termed as OligoR, dedicated to data processing of HDX/MS data of oligonucleotides, spanning all steps from raw MS import to the generation of publication-quality figures. We demonstrate here the use of OligoR with both RT- and CF-HDX/MS data, including bimodal distribution deconvolution. We also exemplify the use of OligoR for native MS kinetics experiments.

## Materials and Methods

Materials and experimental methods are given in the Supporting Information.

### Raw Data File Handling

Raw MS data files in the .raw format (proprietary file format from Thermo) or the .d format (Agilent) were converted to mzML,^38−40^ using the MSConvert utility (Version: 3.0.20175),^41,42^ using the following parameters: 64-bit binary encoding precision, write index, TPP compatibility, zlib compression, and packaged in gzip. All data files are available as demo data alongside the source code; their *m*/*z* and time ranges were filtered with MSConvert to limit their size. Import of raw data in the mzML format in OligoR relies on the mzR R package.^43^

### HDX Calculations

Data were processed in R using the tidyverse^44^ and data.table^45,46^ packages.

#### Deuterated Isotopic Distribution Calculation

The calculation of deuterated isotopic distributions of an oligonucleotide requires the convolution of its natural isotopic distribution with the deuteron distribution. OligoR extracts the chemical composition of the analyte from its sequence and charge state and adds user-supplied atoms (cations and small molecules). The isotopic masses and abundances are those of the last IUPAC Technical Report.^47^ The number of exchangeable sites *nX* of a given oligonucleotide anion is a constant given by eq 1, where *n_dX_* is the number of deoxynucleotides *X*. By default, phosphates are not counted because they are completely deprotonated in the solution and then partially protonated (but not deuterated) by water vapor in the MS source, regardless of the deuterium content in the solution.^36^ Terminal hydroxyl groups are also not accounted for by default.^36^ However, users can choose to account for phosphates and/or hydroxyl groups as needed and can also manually set up *nX* for applications with non-standard oligonucleotides or other types of analytes.
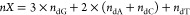
1

The deuterium distribution follows the binomial law, where the number of exchangeable sites (here, amino and imino protons from nucleobases) is the number of trials and the deuterium molar fraction the probability of success. The convolution of natural isotopic and deuteration distributions was performed by fast Fourier transform.^48^ All calculations above were implemented in OligoR based on the code we described previously.^36^

#### Number of Protected Sites and Exchange Rate Determination

The centroid mass-to-charge ratio *m*/*z*_centroid_ is computed from the *n* data points of each distribution using eq 2, where *m*/*z_i_* and *I_i_* are, respectively, the mass-to-charge and intensity of data point *i*.
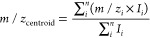
2

Centroids are converted to the number of unexchanged sites NUS as a function of time *t*, calculated using eq 3, where *m*/*z_t_* is the centroid at an exchange time *t*, *m*/*z*_∞_ is the centroid of the fully exchanged reference, and *z* is the charge state of the analyte. The apparent isotopic mass shift for a single site Δ*m*_s_ is defined in eq 4, where *DC*_0_ and *DC*_∞_ are the deuterium content (expressed as molar fractions) in the bulk medium before (*t* = 0) and after (*t* = ∞) mixing, and *m*_D_ and *m*_H_ are the isotopic masses of hydrogen (1.007825 u) and deuterium (2.0141018 u), respectively. The fully exchanged reference can be determined in OligoR from experimental data or the computed theoretical distribution (see above).
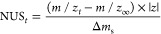
3
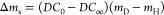
4

The determination of apparent exchange rates by non-linear fitting is performed with eq 5, where NUS_∞_ is the offset, *N_i_* is the number of exchanging sites, *k_i_* is the exchange rate, and *j* is the number of groups of sites with similar rates.
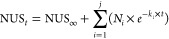
5

The fitting parameters are initialized by linearization of the data and linear fitting, or manually by the user.

#### Modeling and Deconvolution of Isotopic Distributions

The modeling of isotopic distributions is carried out by optimizing for each time point the apparent deuterium content DC of theoretical isotopic distributions (as described above) to the user-selected isotopic distributions. The number of exchangeable sites *nX* is known and kept constant to ensure that the position and width of the optimized isotopic distributions are correct.^49^ The algorithm systematically performs the modeling with one or two distributions; the determination of the effective number of distributions is done afterward by visual inspection and statistical testing.

Theoretical isotopic distribution calculation generates a list of *i* model peaks with their respective *m*/*z_i_* and intensity *I*_model(*i*)_ for a given DC. User-selected distributions are peak-picked with a function based on the peakPick R package,^50^ also generating a list of *i* peaks of intensity *I*_exp(*i*)_ (Figure S1). The *m*/*z* values are rounded to the nearest multiple of 1/*z* to account for slight differences between the *m*/*z* scale of the experimental and model data.

To fit the modeled isotopic distributions to the peak-picked experimental data, the DC variable in the model is optimized by least-squares minimization with the box-constrained L-BFGS-B method.^51^ For each optimization iteration, the sum of square residuals *S* is calculated from the modeled intensities of peaks *i* using eq 6. For bimodal distributions, the optimization is performed using two model distributions *j*, from which a single overall peak intensity *I*_bimodal(*i*, *j*)_ is calculated for each peak *i* for the optimization steps (eq 7). Two optimized DC_*j*_ and two *ab_j_* values are therefore obtained, and two individual distributions are generated from these optimized parameters. The upper and lower DC bounds are the actual initial (DC_*i*_, e.g., 90%) and final (DC_f_, e.g., 9%) experimental DC in the experimental bulk medium, thus avoiding physically impossible results (Figure S2).
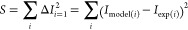
6
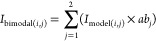
7

There is a risk that significantly overlapped bimodal distributions look monomodal.^37^ Significant overlap happens when EX2 and EX1 occur at the same time, particularly if the number of sites involved in EX1 is small,^37^ and/or if significant exchange through EX2 already occurred (see longer mixing times in Figure S3). Visual inspection alone may not be sufficient to assess this, leading to false-negative detections. Conversely, in the cases where a single population is sufficient, bimodal distributions may still appear to fit marginally better (i.e., lower RSS) because of the larger number of parameters. This could lead to false-positive detections. Therefore, the statistically better model is identified in OligoR by calculation of the *p*-value of the F-statistic defined in eq 8, where *df* is the degree of freedom and mono and bi refer to the mono- and bimodal models, respectively. The null hypothesis is that the bimodal model does not explain the variance better than the monomodal model. The significance level α is set to 0.05 by default and can be changed by the user.
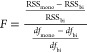
8

#### Derived Parameters

Several HDX parameters are derived from the deconvoluted bimodal distributions. The NUS_*j*_(*t*) values at a time *t* for a population *j* (1 or 2) are obtained from eq 9, where DC_*j*_(*t*) is the optimized DC for the corresponding time and population. This gives access to the “pure” EX2 contributions of each population. These NUS as a function of the exchange time can be fitted using eq 5, similarly to the non-deconvoluted data.
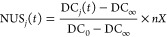
9

Plotting the abundance of each population as a function of the exchange time gives access to the EX1 contribution.^37^ The apparent rate of this contribution, which approximates that of the opening rate of the analyte, is obtained using eq 10, where *ab*_∞_ is the final abundance and *ab*_Δ_ the amplitude of the change of abundance.
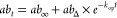
10

### Application Infrastructure, User Interface, and Data Output

#### Application Infrastructure

OligoR is a Shiny application that runs in a web browser.^52−55^ Both the user interface (UI) and server were developed in R. OligoR can be run directly from the source code: it requires the installation of R (version >4.0.5), while package dependencies are automatically installed the first time the application is run. OligoR is also available as a Docker container running R 4.2.1 on Ubuntu 20.04.4 LTS, which only requires to install Docker desktop. This approach allows running OligoR in a predictable way, without potential dependency issues, on any host local computer or cloud.

#### User Interface

The interface is divided into six interconnected modules dedicated to specific tasks.

1. **OligoRef** computes the chemical formula, electrospray series (monoisotopic and average masses and *m*/*z* as a function of *z*), and (deuterated) isotopic distributions of oligonucleotide anions. To do so, the user inputs the sequence, molecularity, and charge state of the analyte. It is also possible to manually add atoms and charges accounting for cation adducts (K^+^ and NH_4_^+^), ligands, or modified nucleotides. The number of exchangeable protons is determined automatically or can be manually specified, which is useful for, e.g., non-canonical nucleotides. Users must also specify the final deuterium content in the solution DC_∞_, which OligoR will automatically take into account when calculating the isotopic distributions. In HDX experiments, DC_∞_ is a critical parameter to (i) constrain the fitting algorithm and (ii) calculate derived parameters (eqs 3, 4, and 9). Experimental isotopic distributions (imported from *MSxploR*) can be compared to the calculated ones to assess the accuracy of the measurement. *OligoRef* also allows determining the number of exchange sites from experimental isotopic distributions, provided that the deuterium solution content is known and the exchange reaction is complete.
2. **MSxploR** imports raw MS data from mzML files, an open format proposed as a community standard.^40^ Users can sum scans, select species/isotopic distributions of interest, and send data to other modules for further processing. For CF-HDX experiments, the scans are combined on a time range wherein the exchange time remains constant and then sent to *MSstackR*. For RT-HDX experiments, wherein each scan corresponds to a different exchange time, all scans are sent to *TimeR*.
3. **MSstackR** displays spectra from discrete exchange time points. It performs isotopic peak picking and mono-/bimodal isotopic distribution modeling and calculates the overlap coefficient Δ. Users can modify the optimization parameters and constraints to fit the experiment and perform statistical testing on the number of distributions. Previously processed data can be loaded as .xlsx files and merged with new data where necessary.
4. **TimeR** filters and plots time-dependent data from RT-HDX experiments or conventional kinetics. It calculates the raw intensity, standard-corrected intensity (if an internal standard is used), and centroid for each scan. Scans can be averaged across a user-supplied scan range if the signal intensity from single scans is deemed insufficient.
5. **HDXplotR** plots HDX data from *MSstackR* and *TimeR* and performs non-linear fitting of exchange kinetics. If bimodal distributions were deconvoluted in *MSstackR*, *HDXplotR* plots the deconvoluted *NUS* and isotopic population abundances and performs non-linear fitting on both.
6. **TitratR** determines amounts of bound and unbound species in equilibrium titrations, from species selected in *MSxploR*, and calculates response factors and dissociation constants by internal standardization. It is a direct implementation of a previously published method that will not be discussed here.^56^

#### Data Output

**Figure 1:**
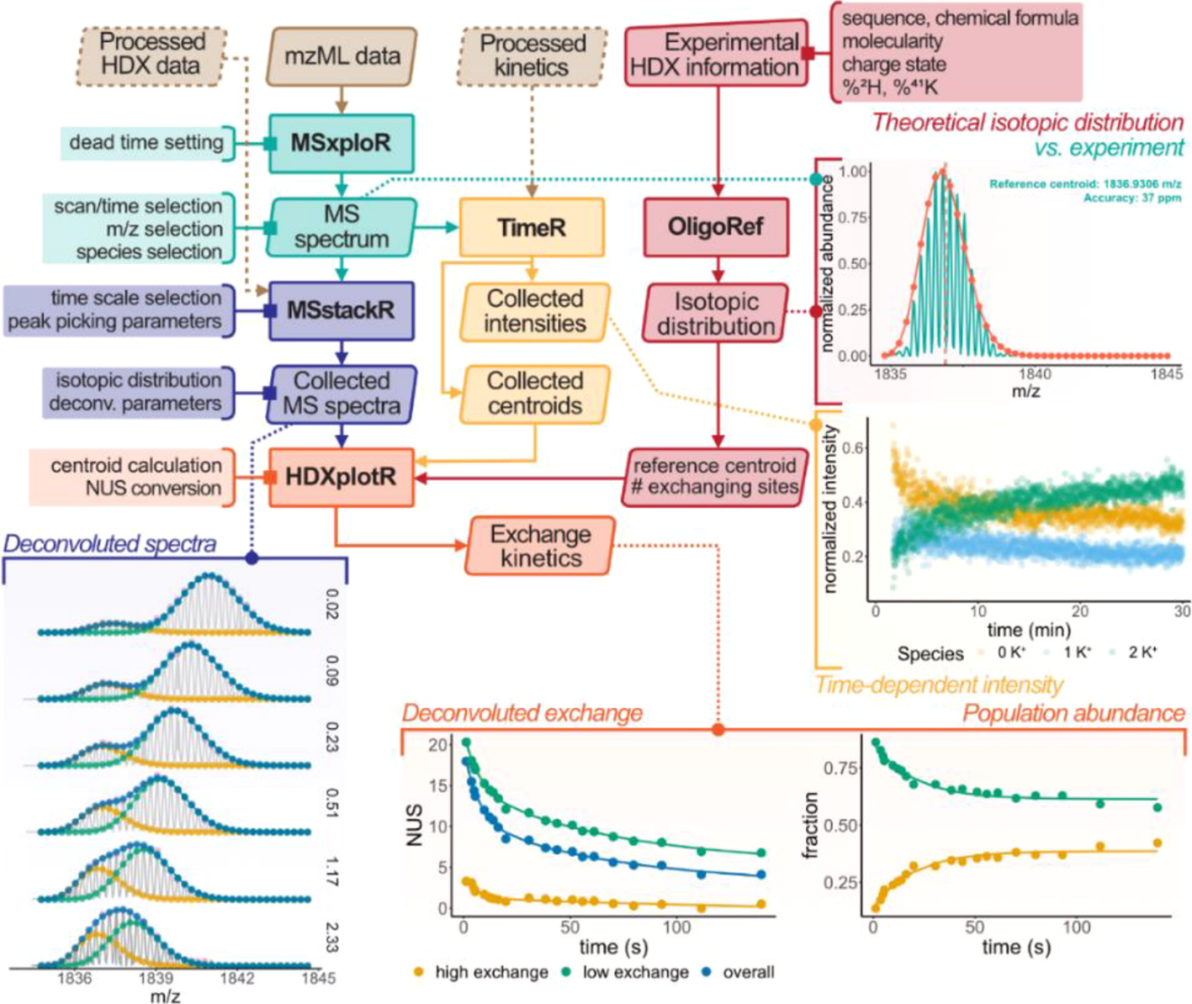
OligoR workflow with main parameters (solid lines with square endpoints) and visualization outputs (dotted lines withcircle endpoints). Input data are shown in brown; dashed input is pre-processed data exported as an .xlsx file from a previous session. Processed HDX data are made of two files (peak-picked and deconvoluted MS spectra). Only selected time points are shown for deconvoluted mass spectra (see Figure S4 for all time points). Visualization outputs were generated with OligoR from CF-HDX data of 23TAG and the binding kinetics of K^+^ with 23TAG (time-dependent intensity output).

Figures are produced using ggplot2 and add-ons.^57−62^ All figures can be customized (e.g., dimensions and colors). Figures can be exported as png or pdf files (for vectorial graphic post-processing). All plots in Figures 1 and 4 were produced with OligoR.

All raw and processed data are displayed in tables built with the *datatables* plug-in for the jQuery JavaScript library. They feature search fields, column sorting and filtering, column and row re-ordering, and export buttons. Data tables can be copied into the clipboard or exported as csv and Excel files. Processed data in the .xlsx format can be re-imported in OligoR. Processed data files for 23TAG, T30177-TT, and VEGF were all made available as demo data alongside the OligoR source code.

## Results

### Isotopic Distribution Deconvolution

The isotopic distribution modeling algorithm was evaluated using CF-HDX data from three oligonucleotides, namely, T30177-TT (5′-T_2_GTG_2_(TG_3_)_3_T), VEGF (5′-CG_4_CG_3_C_2_T_2_G_3_CG_4_T), and 23TAG (5′-TA(G_3_T_2_A)_3_G_3_). T30177-TT is a stable G-quadruplex, whereas VEGF and 23TAG fold into less stable (i.e., more prone to unfold) structures, in our experimental conditions.^63^

**Figure 2:**
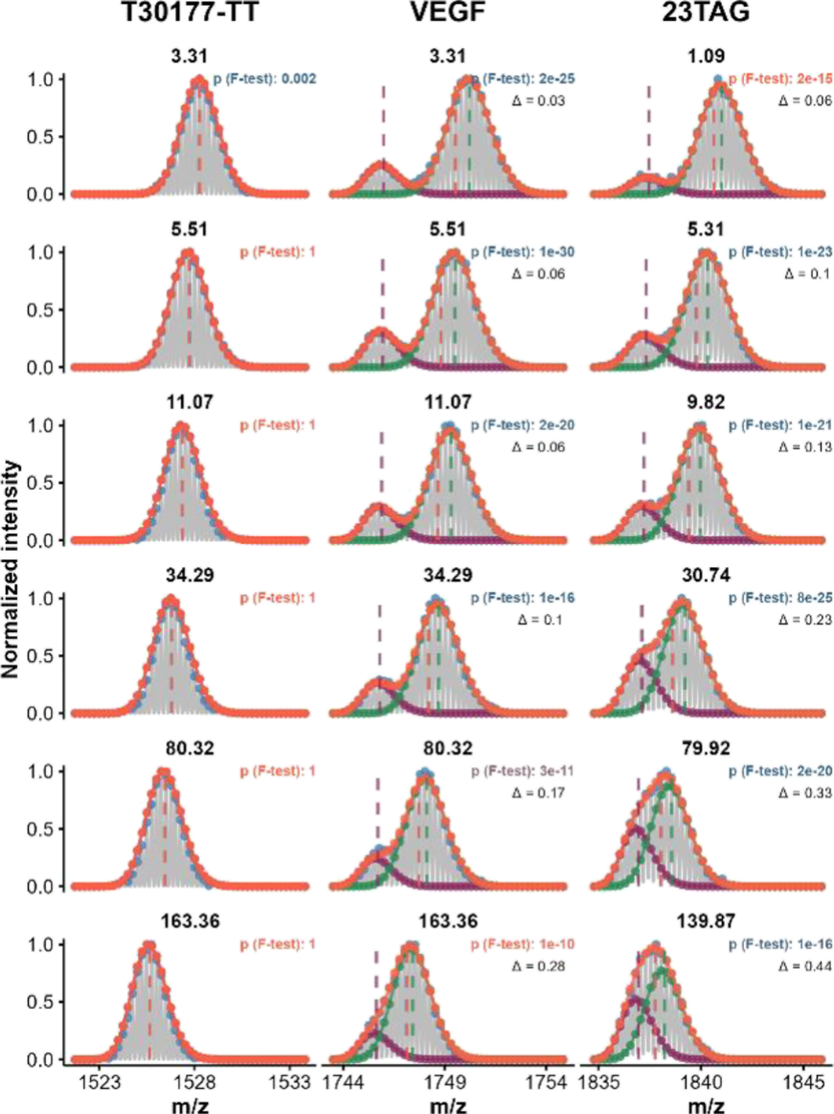
Example of isotopic distribution modeling with OligoR on monomodal and bimodal distributions. The experimental data are shown in gray, peak picking in blue, the overall fit in orange, and individual populations in green and purple (*z* = 4-, K^+^ = 2, time labeled in seconds above panels). The vertical dashed lines show the position of their respective centroids. All time points are shown in Figures S4 and S5. In the case of 23TAG, the abundance of the highly exchanged isotopic population increases, suggesting the presence of EX1 kinetics.^21,37^ On the contrary, there is no change in the abundances of isotopic populations of VEGF; thus, the bimodal distribution probably results from distinct conformations in the solution.

T30177-TT exchanges through an EX2 kinetics characterized by a single isotopic distribution shifting progressively with the exchange time. Thus, a single distribution suffices to fit all time points of T30177-TT (Figure 2). The mean relative difference between the experimental and modeled centroids across the 20 time points is 13 ppm. Conversely, VEGF and 23TAG visibly exhibit bimodal isotopic distributions for most of the time points. At longer mixing times, however, it is not possible to conclude from visual inspection alone whether a single or two highly overlapped isotopic distributions are present. Fitting was achieved with two distributions for all time points, with a mean relative difference in centroid *m/z* of 12 and 10 ppm, respectively. The p-values of the F-statistic indicate that the use of two isotopic distributions is indeed necessary for all time points, for both VEGF and 23TAG, with a high level of confidence (α = 0.05).

**Figure 3:**
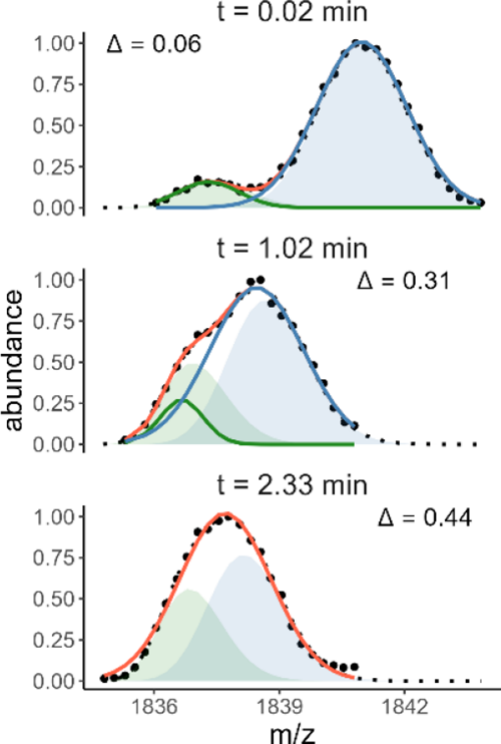
Comparison of fitting methods of increasingly overlapped experimental isotopic distributions (black dots: peak-piked data from 23TAG at three different time points). Gaussian distributions are shown in solid green and blue lines (overall fit in solid orange), and optimized isotopic distributions obtained in OligoR are shown as green and blue areas (overall fit in dotted black). At 2.33 min, only a single Gaussian could be fitted. The coefficient of overlap between the isotopic distributions is given by Δ. The Gaussian fitting and coefficient-of-overlap calculation methods are given in the Supporting Information.

Fitting with Gaussians is a simpler approach that has already been used for protein and peptide isotopic distributions.^20,64^Figures 3 and S3 illustrate the differences between our approach and Gaussian fitting. The latter generally provides an overall fit of the data similar to that of OligoR. However, OligoR automatically provides constraints on the distribution widths based on experimental parameters, whereas a simple Gaussian fitting does not (it would require the determination of peak widths in *m/z* units for any given centroid). Consequently, the individual distributions obtained by Gaussian fitting are wider or narrower compared to isotopic distributions modeled by OligoR, resulting in both incorrect centroids and abundances of the deconvoluted populations. The error increases as the two isotopic distributions become more overlapped, consistent with a previous report.^37^ For instance, for 23TAG, the error is small at 0.02 min but becomes clear at 1.02 min. At 2.33 min, the overlap becomes too important for successful fitting with two Gaussians (i.e., it did not converge) but was achieved with OligoR. A single Gaussian actually fits the experimental data well but is unrealistic: the distribution is too wide for this analyte at this deuteration level, as clearly evidenced by OligoR.

To assess the accuracy of the deconvoluted centroids and abundances as a function of the overlap coefficient, we quantified the fit error on a set of 2181 bimodal spectra generated from predefined deuterium contents for the three sequences T30177-TT, 23TAG, and VEGF at *z* = 4- (Figure S6). Noise was added to better mimic the experimental data, and the relative abundances of the isotopic populations were randomized. The OligoR fitting routine was applied to all spectra (Figures S7–S9). Very accurate centroids were obtained regardless of the overlap coefficient (mean relative error = 0.9%; Figure S10B), with no significant increase in the mean squared error (Figure S10A). The accuracy of the abundances is excellent (mean relative error = 0.9%) for overlaps below 0.5 (Figure S10C), which is the case for all spectra shown in Figure 1. For very large overlap values, however, the relative error increases sharply (4% for 0.5 ≤ Δ < 0.6, 10% for 0.6 ≤ Δ < 0.7, and up to 29% for Δ > 0.7). This is particularly evident for low-abundance populations. Users should therefore be cautious with values obtained from low-abundance, highly overlapping isotope distributions. Such cases are most likely to occur at the very end of exchange kinetics. More generally, users can use Figure S10 as a benchmark to estimate expected accuracies.

### Application of the Workflow

Here, we briefly illustrate the results of CF-HDX, RT-HDX, and binding kinetics experiments using OligoR for data processing and visualization.

#### CF-HDX Processing

We explore here CF-HDX data from experiments involving two species, namely, 23TAG (binding two K^+^) and 23TAG·PhenDC3, its 1:1 complex with the ligand PhenDC3 binding a single K^+^.^65,66^

**Figure 4:**
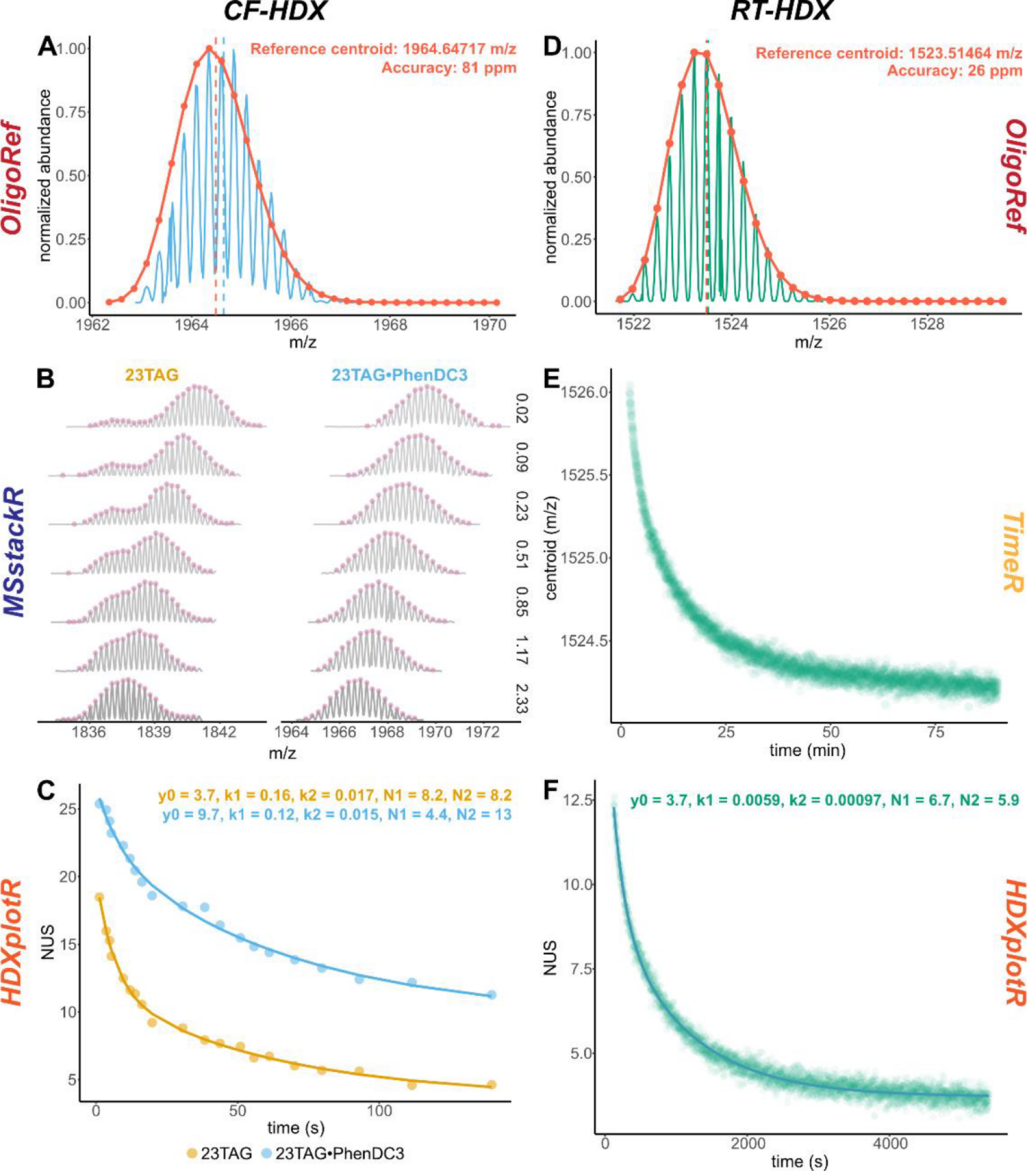
Examples of the use of OligoR to process and visualize HDX/MS results. Left: CF-HDX experiment with two mass-separated species of interest. (A) Empirical determination of the fully exchanged reference centroid (blue line) and comparison to the theory (orange line and points) for 23TAG·PhenDC3 (C_264_H_303_O_141_N_100_P_22_K_1_, *z* = 4- with 51 exchangeable protons, 9%D; 23TAG alone is shown in Figure 1). (B) Collected mass spectra for 23TAG and 23TAG·PhenDC3 for different mixing time points (gray lines; only selected time points shown, see Figure S4 for all time points). Automated peak picking is shown with pink points. (C) Corresponding apparent exchange kinetics (points) and non-linear fitting with eq 5 (lines). Right: RT-HDX experiment acquired for 90 min (dead time: 40 s). (D) Same as (A) for T30177-TT binding two K^+^ (C_190_H_230_O_119_N_74_P_18_K_2_, *z* = 4- with 43 exchangeable protons, 9%D). (E) Calculations of m/z centroids for the species of interest for each of the 3867 scans. (F) Corresponding apparent exchange kinetics for each scan (points) and non-linear fitting with eq 5 (lines).

Figures 1 and 4A show the determination of the fully exchanged reference centroids of 23TAG and 23TAG·PhenDC3, respectively. This is necessary for the calculation of NUS values following eq 3. The *OligoRef* module also compares those experimental data with the theory, which is useful to verify its validity and quantify experimental errors.

Figure 4B displays the isotopic distributions selected in *MSxploR* for each species across seven time points (here, the 4- charge state of 23TAG binding two K^+^, or PhenDC3 and only one K^+^). Peak picking of the isotopic peak, which is necessary for deconvolution, is systematically performed on all collected spectra in *MSstackR*. Note that users can tweak the peak-picking algorithm parameters (e.g., intensity threshold), but default parameters proved very effective in our hands. 23TAG displays bimodal distributions, which are successfully deconvoluted in *MSstackR* (see Figure 1).

*HDXplotR* computes and plots the apparent centroids against the exchange time of both species (Figure 4C), using the references processed in *OligoRef* (Figures 1 and 4A) and experimental parameters provided by the user (e.g., deuterium content and charge states). Non-linear fitting using eq 5 can be toggled on, as shown in Figure 4C.

In the case of unbound 23TAG, *HDXplotR* also plots the deconvoluted exchange and isotopic population abundance as a function of time (Figure 1: low- and high-exchange populations). Non-linear fitting of these two plots, respectively, yields the pure apparent EX2 exchange rate of 23TAG (*k*_1_ = 0.148 s^–1^ and *k*_2_ = 0.014 s^–1^) and the rate of cooperative unfolding undergone by 23TAG to access EX1-capable conformers (*k*_op_ = 0.05 s^–1^).

#### RT-HDX Processing

To illustrate the RT-HDX treatment, we used previously published T30177-TT exchange data.^36^ The fully exchanged reference was obtained in OligoRef, as above (Figure 4D). After selection of the *m/z* range of the isotopic distribution of interest (here, the 4- charge state of the oligonucleotide binding two K^+^) in *MSxploR*, *TimeR* computes the centroid mass for every single scan in a few seconds (Figure 4E). As for CF-HDX, *HDXplotR* converts the kinetics into NUS units and performs non-linear fitting with eq 5 (Figure 4F).

#### Binding Kinetics Processing

We exemplify here the use of *OligoR* for processing binding kinetics using time-dependent data on K^+^ cations binding to 23TAG. This system was first reported by our team to study the folding pathways of G-quadruplexes.^67^

Three species of interest, namely, 23TAG binding 0, 1, and 2K^+^ at the 5- charge state, were selected in *MSxploR*. *TimeR* then readily computes their absolute and relative intensities (shown in Figure 1) as a function of time by processing all scans independently. Here, the abundance of the unfolded strand 23TAG·0K^+^ decreases with time, while the expected equilibrium product, the 3-tetrad G-quadruplex 23TAG·2K^+^, is formed. The 23TAG·1K^+^ ensemble, which includes both off-pathway conformers (2-tetrad G-quadruplex) and folding intermediates, is formed in the first few minutes and is then increasingly converted to the 2K^+^ species.

## Conclusions

HDX/MS is a method of choice to study protein conformational dynamics. Several commercial and academic programs are available for data processing and visualization of the results. None, however, are adapted to the specifics of DNA HDX/MS experiments that we recently introduced. We present here OligoR as an answer to the specific needs of DNA HDX/MS and native MS experiments.

OligoR is divided into six interconnected modules dedicated to specific tasks, from raw data to result visualization and export. Processing of entire experiments spanning many time points can be performed in minutes. Furthermore, we implemented a simple and robust approach to deconvolute bimodal distributions, determine whether they result from different conformers or EX1 kinetics, and access pure EX1 and EX2 exchange rates where relevant. This approach yields physically possible results only and could be expanded to any type of analyte (protein, peptides, sugars, and small molecules) as it relies on chemical formulae, provided that the number of exchangeable sites is known. OligoR provides a means of experimentally determining this number.

All results are presented in data tables that can be exported in several formats to save/reimport data and for other software. Publication-quality figures can be produced, customized, and exported as png or pdf files. Finally, OligoR runs in a simple web browser either directly from the R source code or a Docker container. The latter runs consistently across machines without risks of package dependency or update issues and can be deployed on a server.

The source code and demo data are available on GitHub (https://github.com/EricLarG4/OligoR) and are archived on Zenodo (doi.org/10.5281/zenodo.7691330). The Docker image can be retrieved from the GitHub container registry (ghcr.io/ericlarg4/oligor:master) and Docker Hub (https://hub.docker.com/r/ericlarg4/oligor).

## Supporting Information Available

Additional materials, experimental methods (HDX/MS and native MS kinetics), computational methods (Gaussian fitting and titration experiments), examples of peak-picking results, theoretical isotopic distributions, comparison of binomial and Gaussian fitting, results of isotopic distribution modeling, and evaluation of the influence of the overlap coefficient on the relative fitting error (PDF)
