# Supplementary material for: OligoR: A Native HDX/MS Data Processing Application Dedicated to Oligonucleotides
Source: Anal Chem. 2023 Jun 13;95(25):9615–22. doi: 10.1021/acs.analchem.3c01321 (PMC10308331; doi:10.1021/acs.analchem.3c01321)
Supplement: Supplementary file 1 — ac3c01321_si_001.pdf [file ac3c01321_si_001.pdf]

OligoR: A native HDX/MS data processing application dedicated to oligonucleotides

Eric Largy[1,*] and Matthieu Ranz[1]

[1] Univ. Bordeaux, CNRS, INSERM, ARNA, UMR 5320, U1212, IECB, F-33600 Pessac, France

*To whom correspondence should be addressed.

Supporting Information

Contents

1. Materials S2
2. Experimental methods S2
   2.1. HDX/MS S2
   2.2. Binding kinetics S2
3. Additional computational methods S2
   3.1. Gaussian fitting S2
   3.2. Titration experiments S2
4. Peak picking S3
5. Isotopic distributions S4
6. OligoR vs. Gaussian fitting S5
7. Isotopic distribution modeling S6
8. Influence of the overlap on fit errors S8
   8.1. Generation of bimodal spectra S8
   8.2. Results S9
9. References S13

1. Materials

Trimethylammonium acetate (TMAA) (~1 M aqueous solution) was obtained from Santa Cruz Biotechnology (Heidelberg, Germany), and KCl (99.999% trace metal basis) and D_2O (99.9% D atom) from Sigma-Aldrich (Saint-Quentin Fallavier, France). Samples were prepared in ULC/MS – CC/SFC water (Biosolve Chimie, Dieuze, France).

Oligonucleotides, namely T30177-TT (5’-TTGTGGTGGGTGGGTGGGT), VEGF (5’-CGGGGCGGGCCTTGGGCGGGGT) and 23TAG (5’-TAGGGTTAGGGTTAGGGTTAGGG) were obtained from Eurogentec (Seraing, Belgium) in desalted and lyophilized form, then dissolved in water at around 1 mM.

23TAG was also studied in the presence of a high-affinity G-quadruplex ligand, PhenDC3 (gifted by Dr. Marie-Paule Teulade-Fichou, Université Paris Saclay, Institut Curie, CNRS UMR 9187, INSERM U1196).

2. Experimental methods

2.1. HDX/MS

CF-HDX/MS and RT-HDX/MS data were acquired as previously described, on a Thermo Orbitrap Exactive mass spectrometer operated in negative mode and in soft conditions adapted to native HDX/MS experiments.[1] The samples contained 50 µM DNA in 90% D_2O, 1 mM KCl, 100 mM TMAA (pH = 7.0) and were exchanged with 1 mM KCl, 100 mM TMAA solutions in H_2O (pH = 7.0), yielding 9%-D_2O exchanging solutions.

2.2. Binding kinetics

The binding kinetics of K^+ (500 µM) with 23TAG (10 µM) was recorded on a Thermo Orbitrap Exactive mass spectrometer for 30 minutes. Raw data was converted to the mzML format as described above. Data was analyzed in OligoR by selecting the species of interest (complex with 0, 1 and 2 K^+ for the 5- charge state).

3. Additional computational methods

3.1. Gaussian fitting

Fitting of isotopic distribution with Gaussians was performed with Equation S1, where a_i, b_i and c_i are respectively the area of the peak (reflecting the abundance of the isotopic distribution), position of the center of peak (centroid of the isotopic distribution) and full width at width at half maximum of the peak ($FWHM = 2 \times \sqrt{2\ln(2)} \times \sigma$, where σ is the standard deviation).

$$y = \sum_{i=1}^{2} a_i \exp\left(\frac{(m/z-b_i)^2}{2c_i^2}\right) \qquad \text{(S1)}$$

The estimate of overlap Δ between isotopic distributions was calculated with the overlapTrue function from the overlap R package.[2]

3.2. Titration experiments

The determination of response factors and K_d from titration experiments was implemented in R following our previously published method.[3] The calculation of Moore-Penrose generalized inverse of matrices was implemented using the gnm package.[4]

4. Peak picking

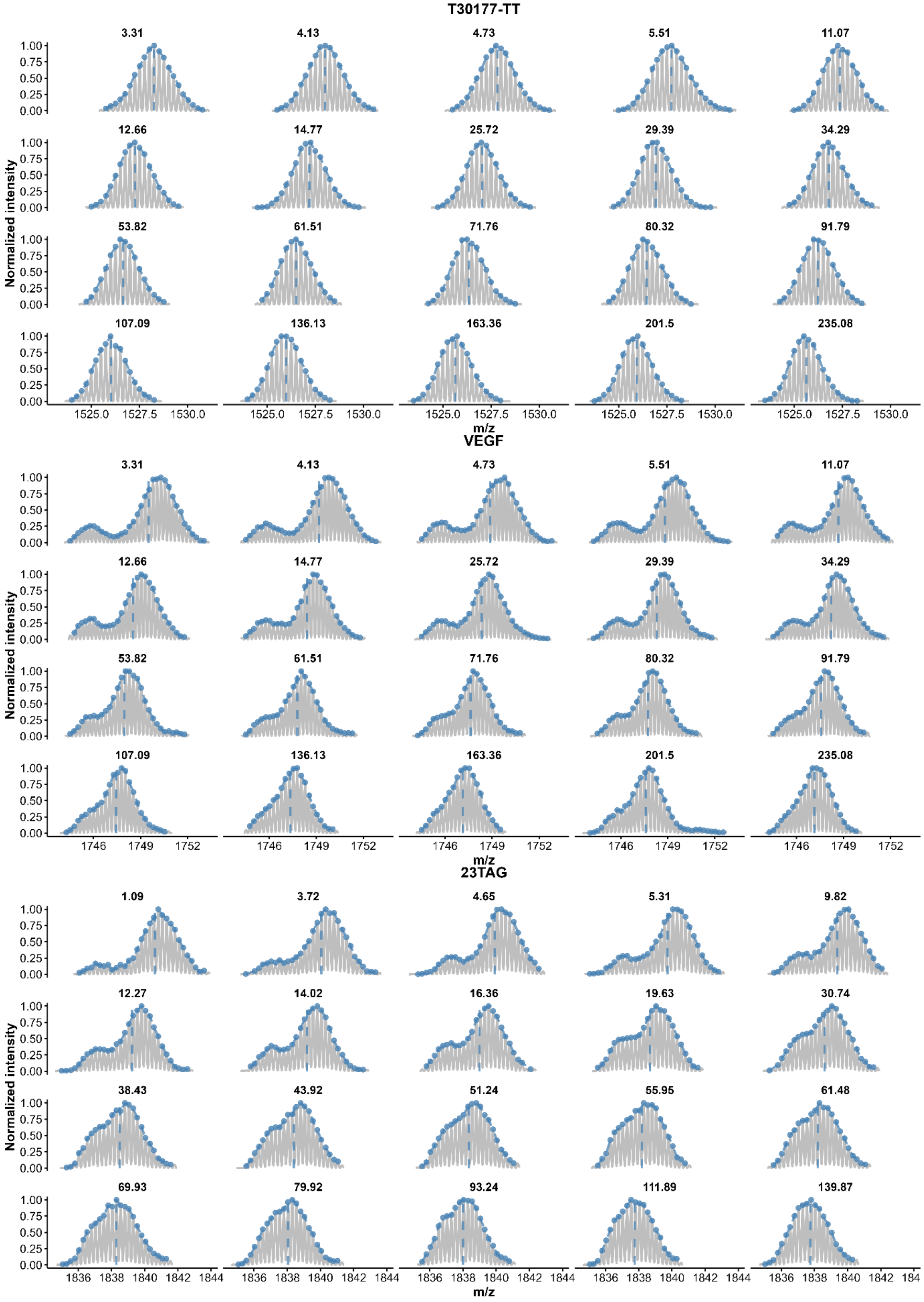

Figure S1. Example of peak picking on the continuous-flow exchange data of T30177-TT, VEGF and 23TAG (z = 4-, K^+ = 2). The experimental data is shown in grey, the peak picking in blue, and the centroids as dashed, blue vertical lines.

5. Isotopic distributions

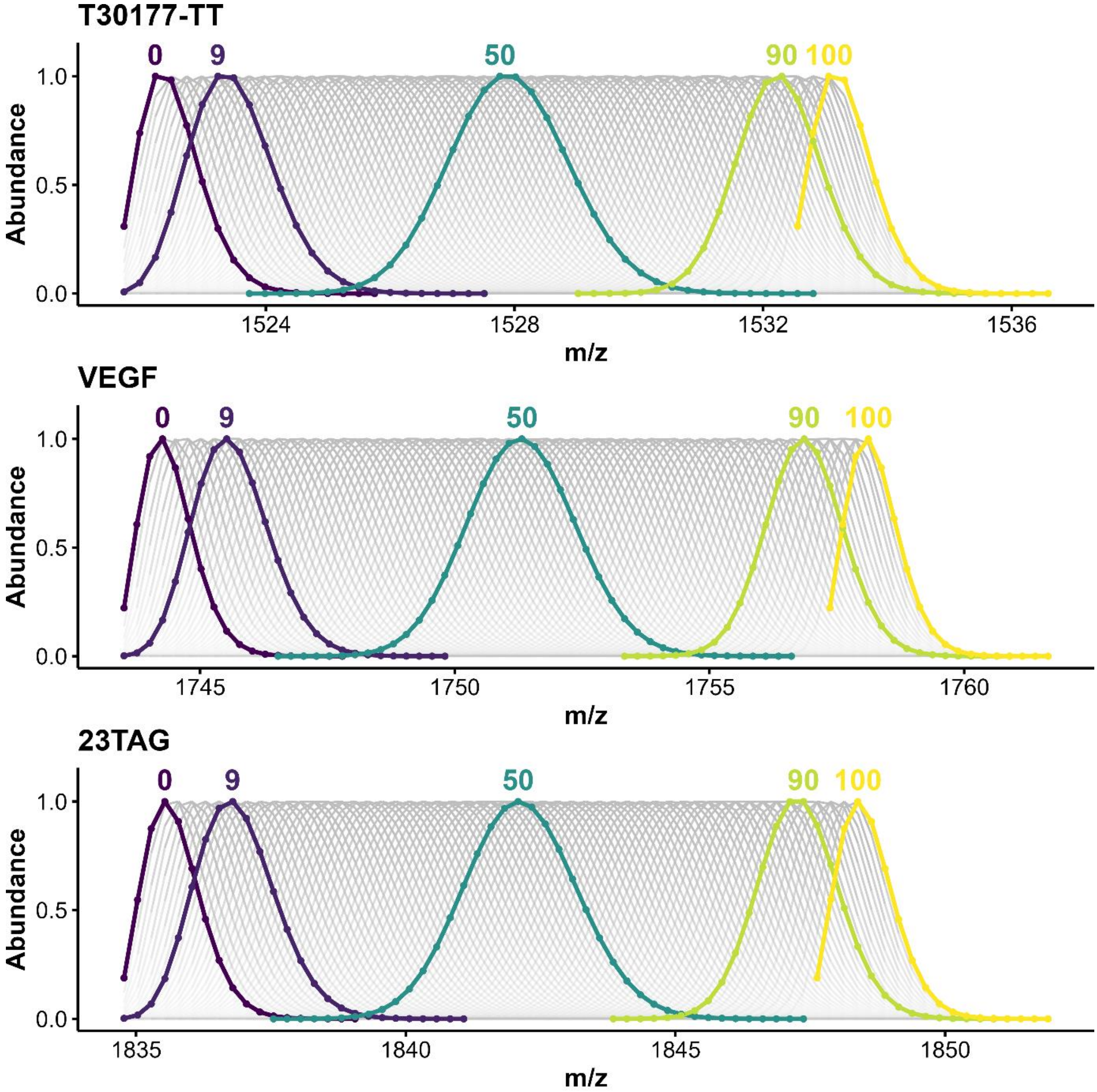

Figure S2. Examples of physically-possible distributions for T30177-TT, VEGF, and 23TAG (z = 4-, K^+ = 2, DC = 0—100%). The grey lines show the range of possible distributions when the deuterium content is varied between 0 and 100% (computed from 101 distributions evenly spaced by 1% deuterium increments; abundances smaller than 10^{-5} were filtered off). Colored lines highlight particular deuterium contents (0, 9, 50, 90, and 100%). Note the difference in widths at half maximum, which is largest at 50% deuterium content and narrowest at 0 and 100%.

6. OligoR vs. Gaussian fitting

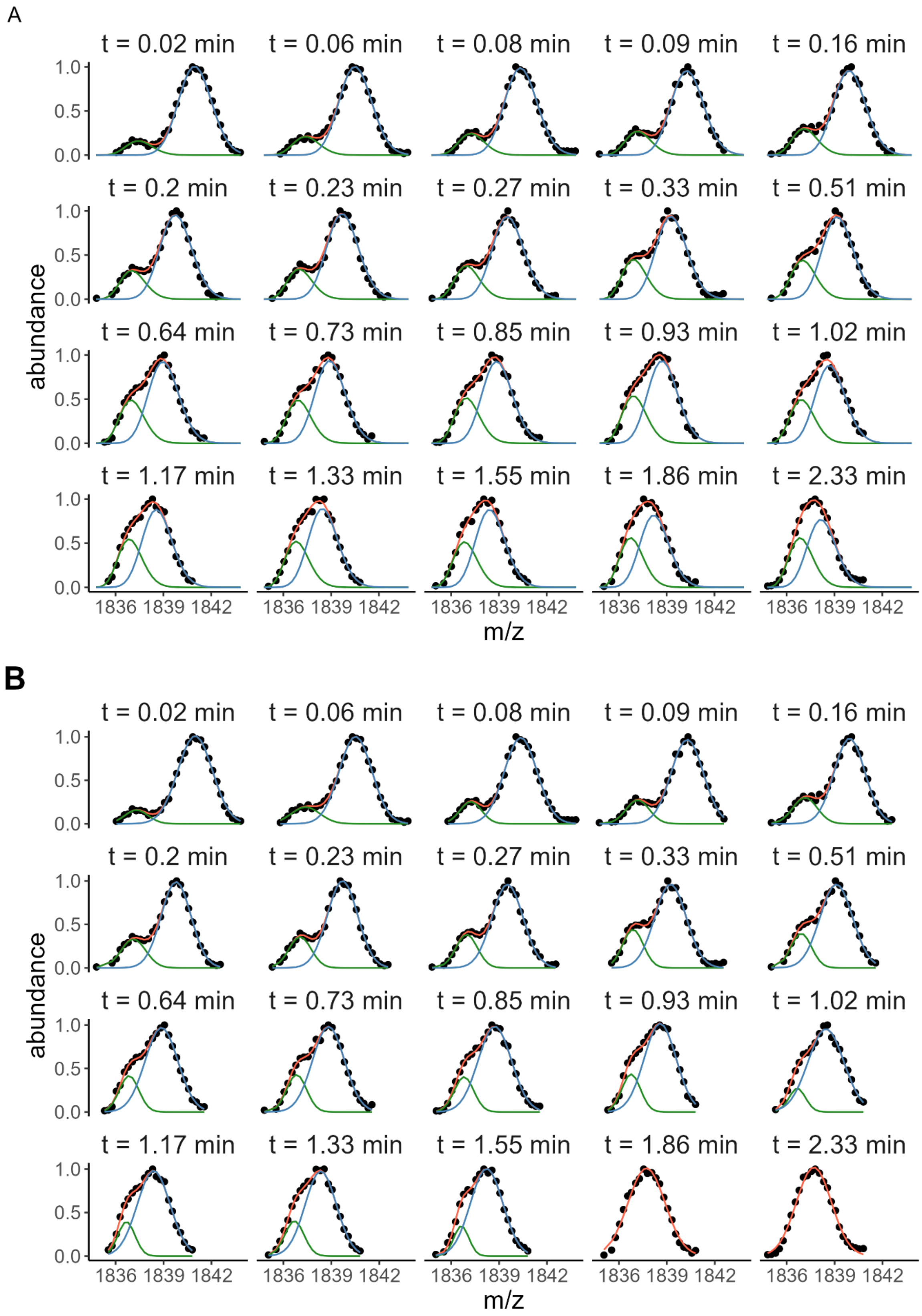

Figure S3. Deconvolution of bimodal isotopic distributions by A. least-square minimization of theoretical distributions or B. Gaussians.

7. Isotopic distribution modeling

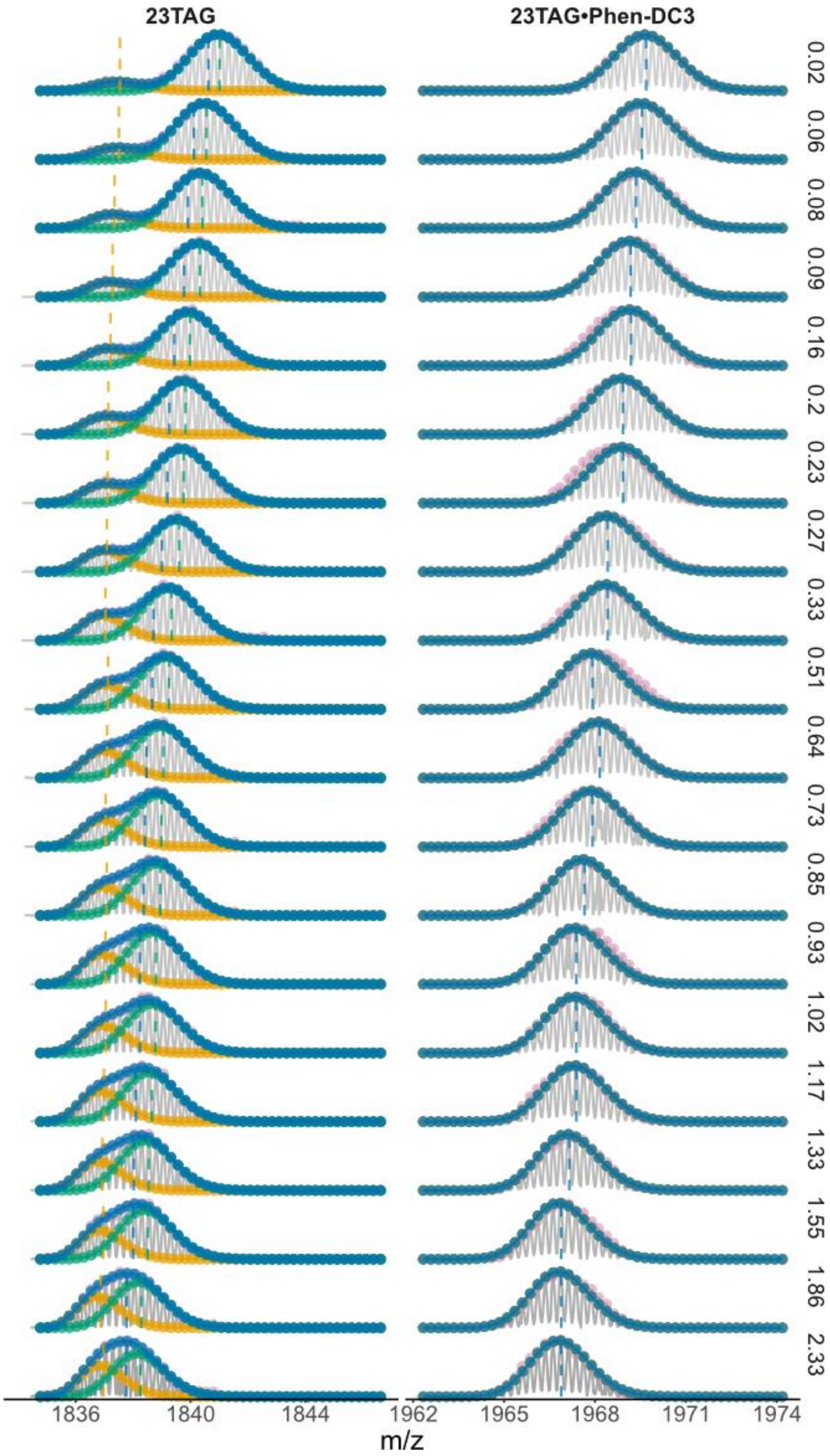

Figure S4. Isotopic distribution modeling with OligoR on bimodal (left; 23TAG, z = 4-, K^+ = 2) and monomodal distributions (23TAG•Phen-DC3, z = 4-, K^+ = 1). The experimental data is shown in grey, the peak picking in pink, the overall fit in blue, and individual population in green and orange, time labeled in minutes.

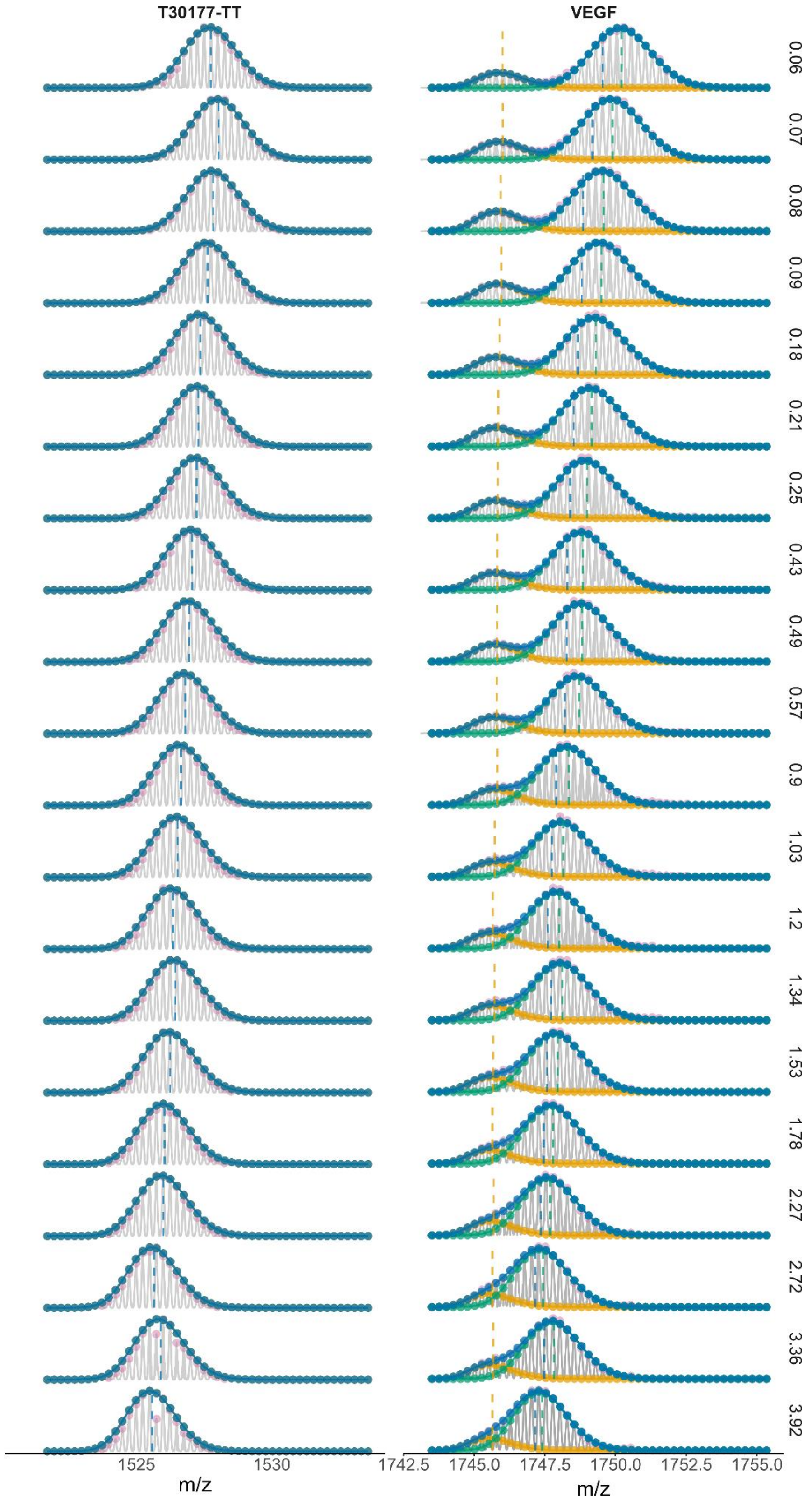

Figure S5. Isotopic distribution modeling with OligoR on monomodal (left; T30177-TT, z = 4-, K⁺ = 2) and bimodal distributions (VEGF, z = 4-, K⁺ = 2). The experimental data is shown in grey, the peak picking in pink, the overall fit in blue, and individual population in green and orange, time labeled in minutes.

8. Influence of the overlap on fit errors

8.1. Generation of bimodal spectra

Bimodal spectra were generated by convoluting two isotopic distributions generated by OligoR, specifically with the function peak.positionR, which takes into account the oligonucleotide sequence, the number of exchangeable sites, and the solution deuterium content. For each bimodal spectrum, the abundances were randomized and the deuterium content DC_i of a population i was set as an integer between 9 and 50 (in %), with $DC_2 > DC_1$ (to discard duplicates) and a maximum difference ($DC_2 - DC_1$) of 30 to only obtain spectra with some degree of overlap. The application of this approach to three sequences T30177-TT, 23TAG and VEGF binding 2 K^+ at z = 4- generated 2181 bimodal spectra with overlaps ranging from 0.03 to 0.94.

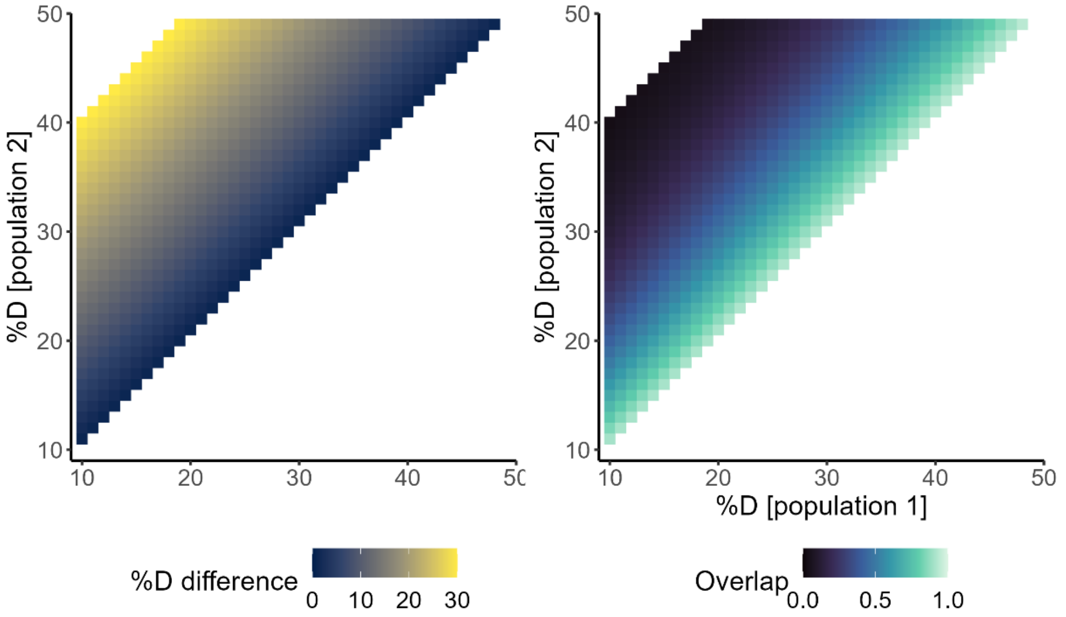

Figure S6. Heatmap of the difference in deuterium content between the two generated populations (left) and corresponding overlaps for $[23TAG{\bullet}2K^+]^{4-}$ (right). Each tile corresponds to a bimodal spectrum, and this approach was applied to three sequences.

Noise was added on the y axis using a random generation function (*rnorm*, from the *stats* package for R) for the normal distribution with the standard deviation set to 0.01.

8.2. Results

The fitting algorithm of OligoR was then applied on all spectra. A few randomly sampled examples are shown below.

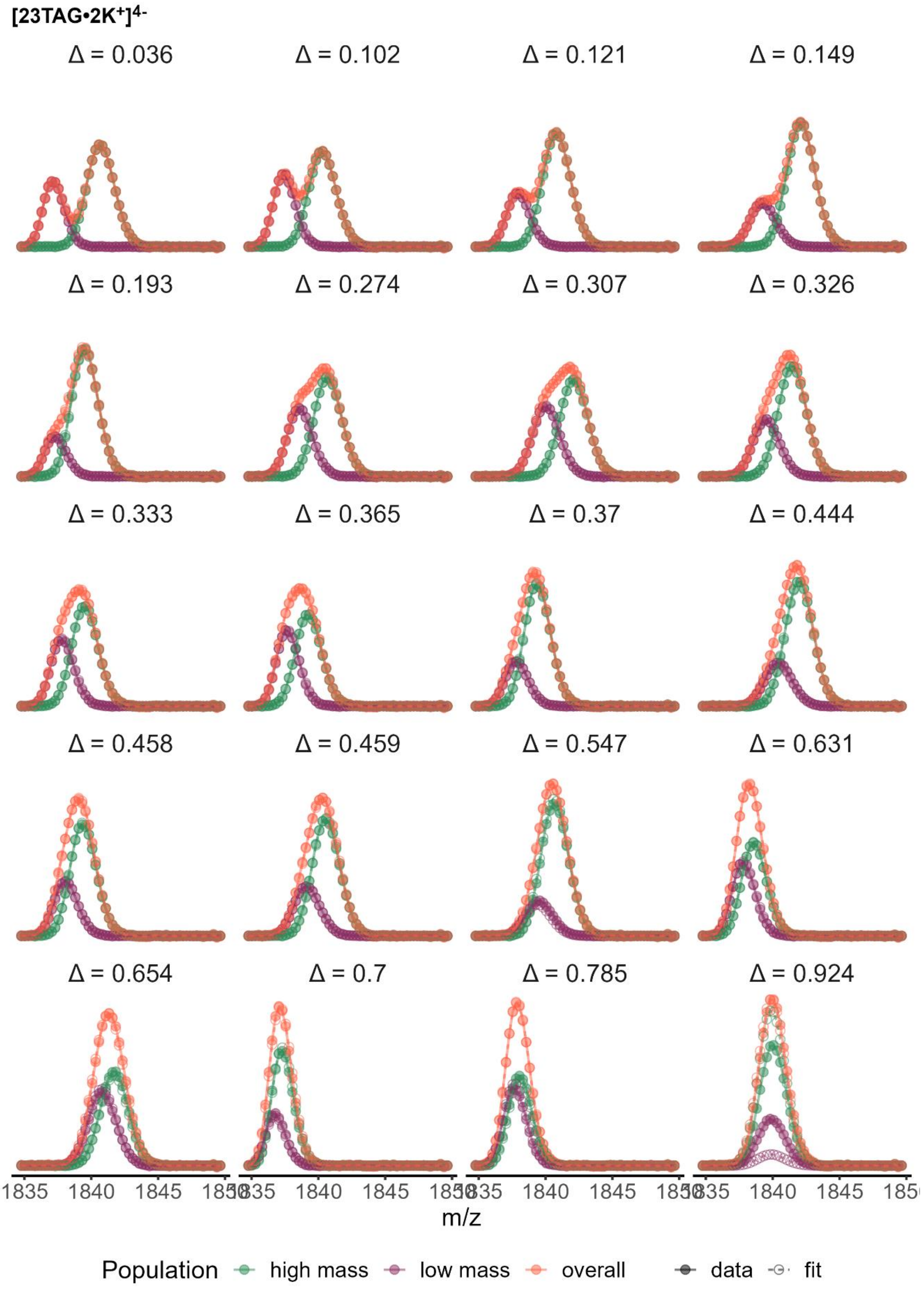

Figure S7. Examples of fitting results for [23TAG•2K$^+$]$^{4-}$ for increasing overlap coefficients Δ. Inaccurate abundances are obtained for Δ = 0.924.

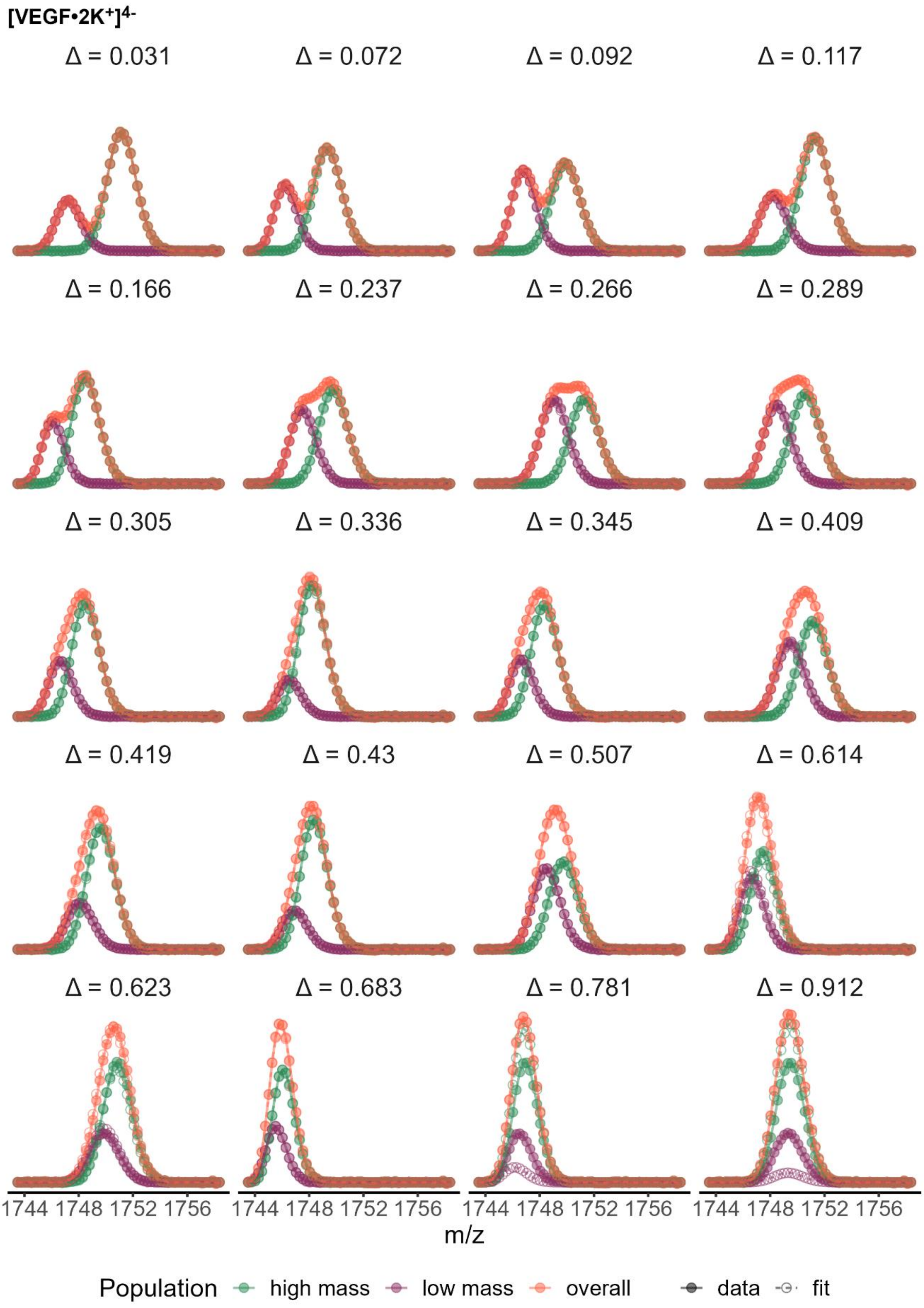

Figure S8. Examples of fitting results for [VEGF•2K+]4- for increasing overlap coefficients Δ. Inaccurate abundances are obtained for Δ = 0.781 and 0.912.

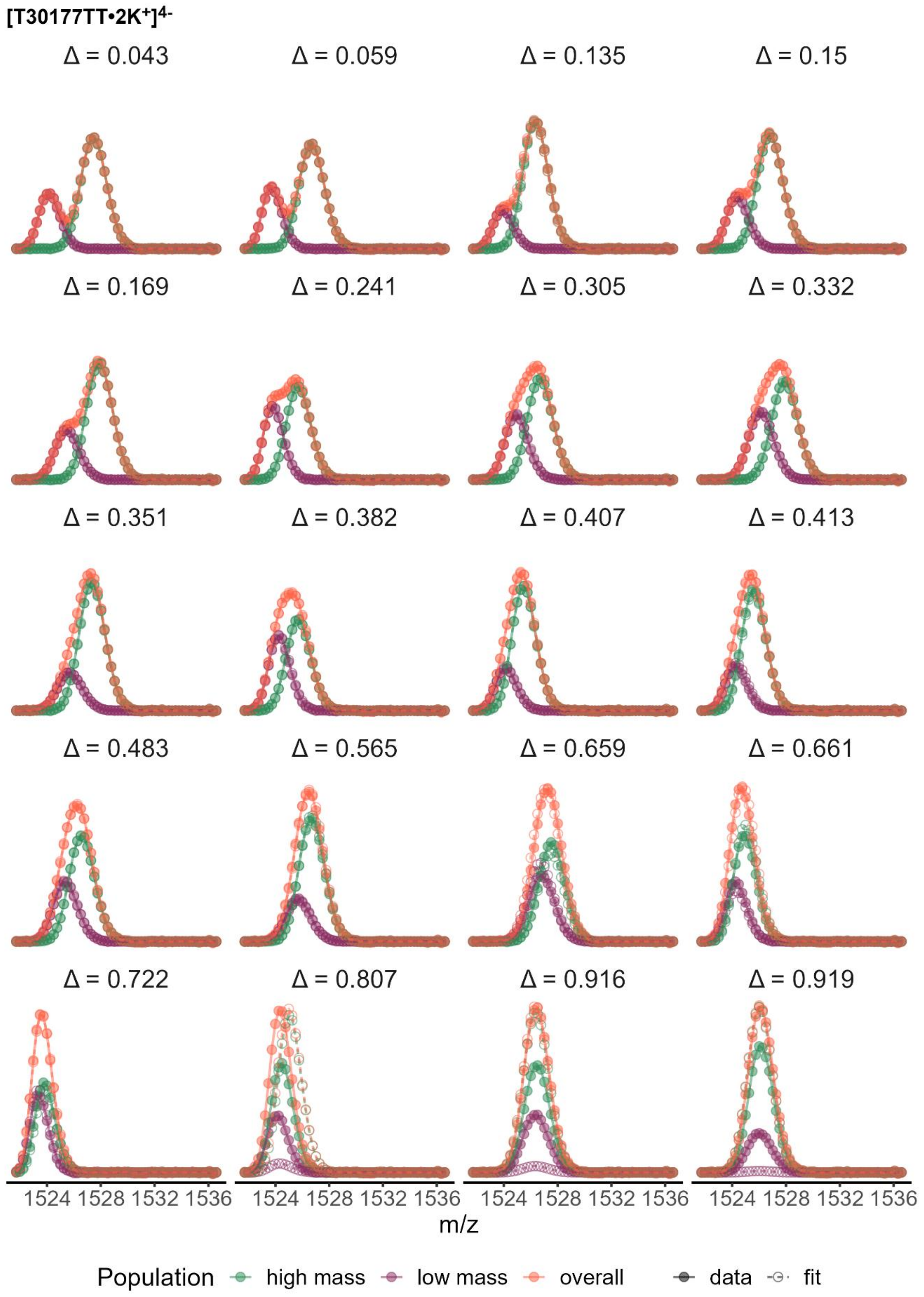

Figure S9. Examples of fitting results for [T30177TT•2K$^+$]$^{4-}$ for increasing overlap coefficients Δ. Inaccurate abundances are obtained for Δ = 0.807, 0.916 and 0.919.

The mean squared error (MSE), centroid error, and isotopic population abundance error were plotted as a function of the overlap between the isotopic population.

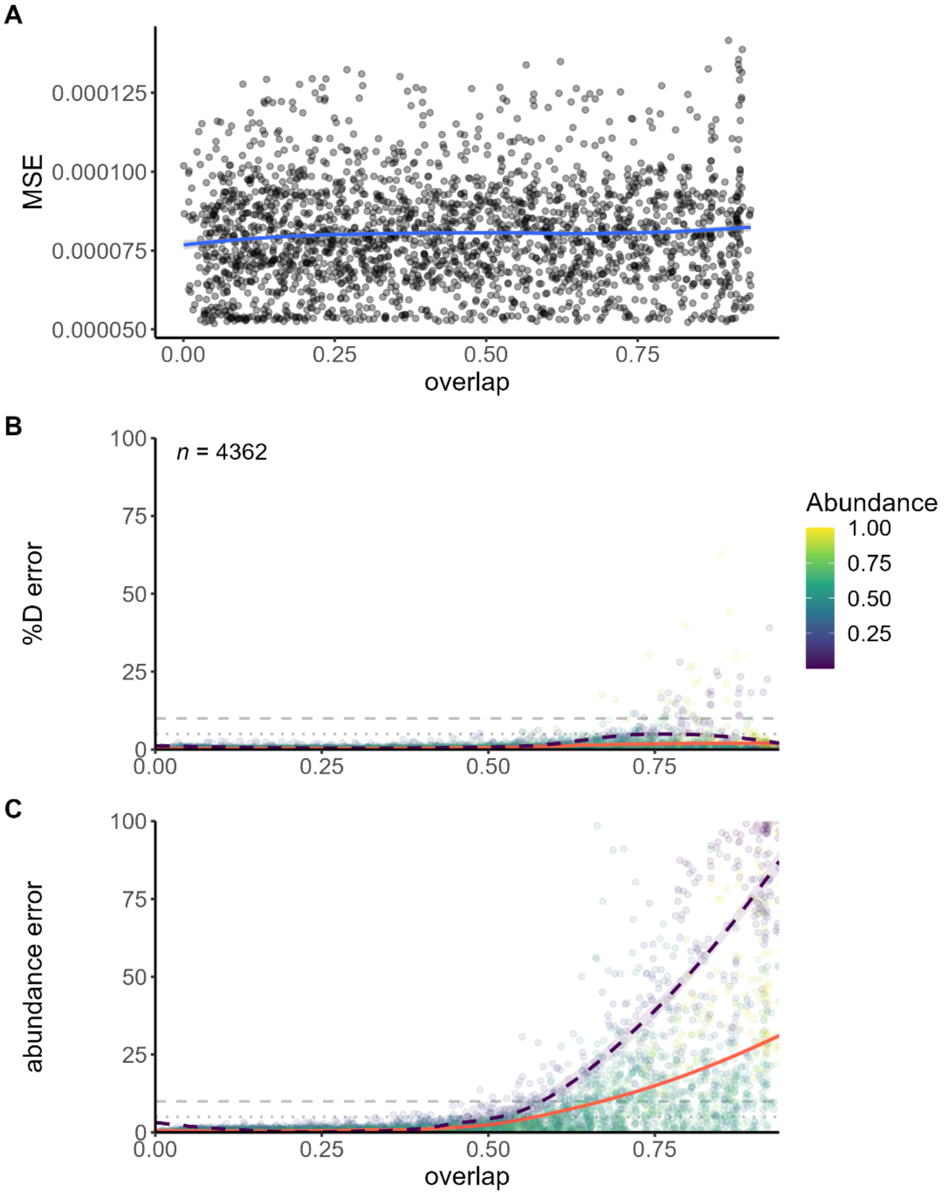

Figure S10. Fitting error as a function of the overlap coefficient. A. Mean squared error. Local regression (LOESS method; blue line) does not show a significant increase of the MSE. B. Centroid error. The mean relative error is below 1%, with few points above 5% (dotted line) and 10% (dashed line) error. Local regression on all data (LOESS; orange line) and populations with relative abundance below 0.25 (dashed purple line) shows that the error tends to be larger for low abundant populations, but only at high overlap values. C. Abundance error. The mean relative error is below 1% for overlaps < 0.5 but increases for larger overlaps (orange line) and in particular for low abundance populations (dashed purple lines).

9. References

(1) Largy, E.; Gabelica, V. Native Hydrogen/Deuterium Exchange Mass Spectrometry of Structured DNA Oligonucleotides. *Anal. Chem.* **2020**, *92* (6), 4402–4410. https://doi.org/10.1021/acs.analchem.9b05298.

(2) Ridout, M. S.; Linkie, M. Estimating Overlap of Daily Activity Patterns from Camera Trap Data. *J. Agric. Biol. Environ. Stat.* **2009**, *14* (3), 322–337. https://doi.org/10.1198/jabes.2009.08038.

(3) Gabelica, V.; Rosu, F.; Pauw, E. D. A Simple Method to Determine Electrospray Response Factors of Noncovalent Complexes. *Anal. Chem.* **2009**, *81* (16), 6708–6715. https://doi.org/10/c42m5f.

(4) Turner, H.; Firth, D. Generalized Nonlinear Models in R: An Overview of the Gnm Package, 2022. https://cran.r-project.org/package=gnm.
